# Integrated Serum and Fecal Metabolomics Study of Collagen-Induced Arthritis Rats and the Therapeutic Effects of the Zushima Tablet

**DOI:** 10.3389/fphar.2018.00891

**Published:** 2018-08-14

**Authors:** Jinjun Shan, Linxiu Peng, Wenjuan Qian, Tong Xie, An Kang, Bei Gao, Liuqing Di

**Affiliations:** ^1^Jiangsu Key Laboratory of Pediatric Respiratory Disease, Institute of Pediatrics, Nanjing University of Chinese Medicine, Nanjing, China; ^2^Medical Metabolomics Center, Nanjing University of Chinese Medicine, Nanjing, China; ^3^State Key Laboratory Cultivation Base for TCM Quality and Efficacy, School of Pharmacy, Nanjing University of Chinese Medicine, Nanjing, China; ^4^Jiangsu Key Laboratory for Functional Substance of Chinese Medicine, Nanjing, China; ^5^Genome Center of UC Davis, NIH West Coast Metabolomics Center, Davis, CA, United States

**Keywords:** metabolomics, rheumatoid arthritis, Chinese medicine, gut microbiota, gas chromatography-mass spectrometry

## Abstract

The Zushima tablet (ZT) has been used for decades in the clinical treatment of rheumatoid arthritis (RA) in China. However, its therapeutic mechanism is unclear. In this study, we aimed to explore the distinctive metabolic patterns in collagen-induced arthritis (CIA) rats and evaluate the therapeutic effects of ZT on RA using untargeted serum and fecal metabolomics approaches based on gas chromatography coupled with mass spectrometry. Body weight, hind paw swelling, TNF-α and IL-1β levels, arthritis scores, and histopathological parameters were assessed. In the metabolomics study, 31 altered metabolites in the serum and 30 in the feces were identified by comparing the model with the control group using statistical processing. These altered metabolites revealed that the tricarboxylic acid cycle, glycolysis metabolism, fatty acid metabolism, and purine metabolism were disturbed in CIA rats, and most of these altered metabolites including l-isoleucine, l-aspartic acid, pyruvic acid, cholic acid, and hypoxanthine, were rectified by ZT. Furthermore, short-chain fatty acids in feces were quantitatively determined, and the results showed that ZT could regulate the levels of propionate, butyrate, and valerate in CIA rats. Then, gut microbiota were analyzed by 16S rRNA analysis. Our results showed that *Firmicutes* and *Bacteroidetes* were the most abundant bacteria in rats. The levels of 19 types of bacteria at the family level were altered in RA rats, and most of them could be regulated by ZT. This study demonstrated that metabolomics analysis is a powerful tool for providing novel insight into RA and for elucidating the potential mechanism of ZT.

**Graphical Abstrac:**
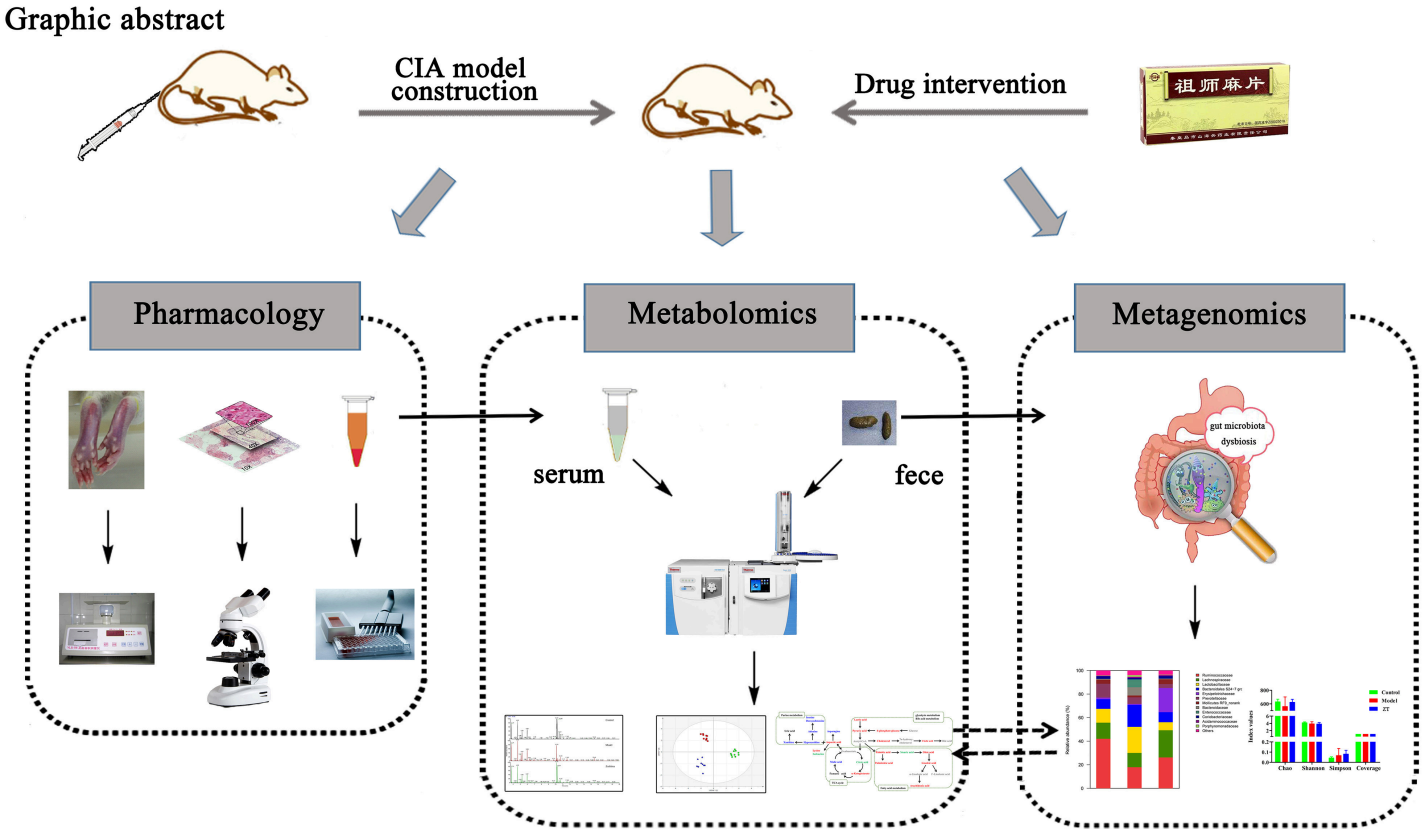
Summary of experiment processes includes pharmacology, metabolomics and metagenomics.

## Introduction

Rheumatoid arthritis (RA) is a chronic, systemic, autoimmune disease accompanied by persistent synovitis (inflammation of the synovial membrane), emergence of cartilage erosion, and destruction of the bone and joints (Qi et al., 2014). It has been reported that RA is always associated with oxidative stress (Kundu et al., 2012) and high levels of cytokines, including interleukins (IL-1) and tumor necrosis factor-α (TNF-α) (Arend and Dayer, 1995). Although many studies have focused on RA, its pathogenesis is still not clear. In recent years, several studies have reported that impairment in the interaction between the gut microbiota and mucosal immune system can lead to inflammatory diseases such as RA. This finding contributed to a better understanding of this complex disease (Cai et al., 2012; Gomez et al., 2012; Li et al., 2015a; Chen et al., 2016).

Owing to its pathogenetic holism and complexity, no specific medicine has been able to effectively cure RA. Disease-modifying anti-rheumatic drugs (DMARDs) and non-steroidal anti-inflammatory drugs (NSAIDs) have improved clinical symptoms in patients with RA (Van Vollenhoven, 2009). However, side effects such as liver and gastrointestinal disorders limit their extensive clinical use (Cavagna et al., 2013; Moller et al., 2015). Herbal medicines, which are widely used in some traditional medical systems, have provided a huge source of new drugs that can be used to treat complicated diseases (Li et al., 2015b).

The Zushima tablet (ZT), a traditional Chinese patented prescription medicine, was officially recorded in the Pharmacopeia of the People's Republic of China (2015 edition) for the treatment of RA. ZT mainly contains coumarins and flavonoids, such as daphnetin, daphnoretin, 7-hydroxycoumarin, yuankanin, daphneticin, and rutarensin. These components were reported to have analgesic, anti-inflammatory, and anti-bacterial activities (Gao et al., 2008; Chen et al., 2011; Huyiligeqi et al., 2016). However, the therapeutic effects of ZT on RA should be investigated further to understand its comprehensive mechanism of action for better clinical application.

Metabolomics is usually applied to understand the function of biological systems based on the global metabolite profiles influenced by pathological stimuli or drug treatments in biological samples (Gu et al., 2015). Generally, serum and fecal metabolomics provide biomarker information associated with physiological or pathophysiological processes (Huyiligeqi et al., 2016; Khamis et al., 2017). Therefore, the combination of the two metabolomics approaches can provide a more comprehensive and detailed holistic metabolic profiling. In this study, serum and fecal metabolomics approaches were carried out using gas chromatography-mass spectrometry (GC-MS) to obtain the metabolites altered by RA and elucidate the regulatory mechanism of ZT. Furthermore, fecal samples were investigated by 16S rRNA sequence analysis to analyze the altered gut microbiota and subsequently explore the association of gut microbiota with endogenous metabolites. The present study aimed to investigate the pathogenesis of RA through global serum and fecal metabolomics approaches, which can aid in the identification of metabolites associated with gut microbiota, and to evaluate the potential protective mechanism of ZT.

## Materials and methods

### Chemicals and reagents

Bovine type II collagen was purchased from Chondrex (Redmond, WA, USA). Acetic acid was purchased from Nanjing Chemical Reagents Co., LTD (Nanjing, Jiangsu, China). Freund's complete/incomplete adjuvant, methoxyamine hydrochloride, pyridine, 1, 2-^13^C-myristic acid, and N, O-Bis(trimethylsilyl)trifluoroacetamide with trimethylchlorosilane (BSTFA) were all obtained from Sigma-Aldrich (St. Louis, MO, USA). Both n-hexane and acetone were purchased from ROE Scientific (St. Louis, MO, USA). Methanol of MS grade was supplied by Merck Millipore (Billerica, MA, USA). ZT was purchased from Shanhaiguan Pharmaceutical Co., Ltd (Qinhuangdao, Hebei, China, batch number: 20161202). The quality of ZT was also evaluated and the method is shown in the Supplementary File. Carboxymethylcellulose sodium (CMC-Na; 300–800 mPa.S) was obtained from Sinopharm Chemical Reagent Co., Ltd (Shanghai, China). Distilled water was purified using a Milli-Q purification system (Millipore, Milford, MA, USA).

### Animals

Thirty-four male Sprague-Dawley rats weighing 180–220 g were supplied by Jiesijie lab animal Ltd. (Shanghai, China), and the animal certification number was SCXK-2013-0006. All rats were fed in an environmentally controlled room with constant temperature (20–24 °C) and humidity (45–60%) of SPF grade with a standard 12 h light/dark cycle for 5 days before the formal experiment. After 5 days of adaptive feeding with free access to water and chow during the experiment, the rats were divided into two groups: 10 rats in the control group and 24 rats for collagen-induced arthritis (CIA) model development. The animal experiments were performed under the guidelines of the Animal Ethics Committee of Nanjing University of Chinese Medicine.

### CIA model construction

The CIA rat model was established according to the Experimental Methodology of Pharmacology (Wu and Li, 2010). Bovine type II collagen (20 mg) was dissolved in 10 mL acetic acid solution (0.05 M) and the mixture (CII-AA) was shaken at 4°C overnight. After 12 h, CII-AA was emulsified with Complete Freund's Adjuvant (CFA) at a ratio of 1:1 using an Ultra-Turrax high speed disperser (IKA®Works Guangzhou, Guangzhou, Guangdong, China) until the CII-CFA emulsion remained unscattered in water. In addition, CII-AA was emulsified with Incomplete Freund's Adjuvant (IFA) to prepare the CII-IFA emulsion using the same protocol mentioned above.

CIA model rats were intradermally (i.d.) injected with 0.2 mL CII-CFA emulsion at the base of the tail (day 1). On day 7 after the primary immunization, the rats were boosted intradermally with 0.1 mL CII-IFA. The normal rats were injected with the same volume of physiological saline.

### Drug administration and sample collection

Almost 2 weeks after model development, the rat models with RA were divided randomly into model and ZT groups. To assess its therapeutic efficacy, ZT was suspended in 0.5% CMC-Na solution, and rats in the ZT group were administered ZT intragastrically (0.6 g/kg, each tablet weighs 0.3 g, 2-fold the clinical dosage) from the day after the onset of arthritis (day 14) and once daily until day 36 of the experiment. In addition, the rats in the control and model groups were treated with the same volume (10 mL/kg) of 0.5% CMC-Na solution.

Serum and fecal samples were collected on day 0 (prior to immunization), 14 (prior to therapy), 21, and 36, respectively. Blood samples were collected from the postorbital venous plexus veins and kept at room temperature for 1 h. Then, the samples were centrifuged at 4,000 rpm for 5 min to obtain the serum. In addition, around 2–3 stool samples were stored in liquid nitrogen immediately after collection. Both serum and feces were stored at −80°C until analysis.

### Basic physiological parameters

During the experiment, three basic physical parameters were selected for evaluating model development and efficacy of ZT: body weight, paw swelling, and arthritis scores. The body weights and paw swelling were assessed every 3–4 days after the rats were injected with collagen. As a significant index for evaluating the seriousness of RA, paw swelling was expressed using the average value of left and right hind paws. Meanwhile, the arthritic scores were recorded from day 12 to 36 at an interval of 4 days. The severity of arthritis was graded according to the following criteria (Zhang et al., 2014): 0 score: no pathological changes; 1 score: slight redness or swelling in the palms; 2 score: slight redness or swelling in the ankle joint and toe joints; 3 score: moderate redness and swelling in the palms, ankle, and toe joints; and 4 score: serious redness and swelling in the palms, ankle, and toe joints. All four paws were assessed, and the values were added for each rat. The highest score for each rat was 16.

### Quantification of serum TNF-α and IL-1β and histopathology analysis

All rats were sacrificed on day 36, and blood was collected to obtain serum for measuring the inflammatory factors TNF-α and IL-1β using ELISA kits (Multi Sciences, Hangzhou, Zhejiang, China). Meanwhile, the right ankle joint of each rat was fixed in formalin for histopathologic analysis.

### Serum and fecal sample preparation for metabolomics analysis

Serum samples were thawed at 4°C, and an aliquot of 50 μL serum was transferred into a 1.5 mL Eppendorf tube. Then, 200 μL methanol (with 12.5 μg/mL 1,2-^13^C-myristic acid) was added to precipitate proteins as well as to mix the internal standards with samples. The samples were centrifuged at 14,000 rpm for 10 min with an Allegra 64R High-Speed Refrigerated Centrifuge (Beckman Coulter, Brea, CA, USA) after vortexing for 3 min using a vortex-genie (Scientific Industries, Bohemia, NY, USA). The supernatant (100 μL) was transferred into a new tube and evaporated to dryness at 45 °C and 15 kPa in a Thermo SPD1010-230 SpeedVac Concentrator (Thermo Fisher, San Jose, CA, USA). After drying, 30 μL of methoxyamine hydrochloride dissolved in pyridine (w/v, 10 mg/mL) was added and vortexed for 5 min. Then, the samples were shaken at a speed of 300 rpm for 90 min in a thermostatic oscillator at 30°C. Subsequently, 30 μL of BSTFA (1% TMS) was added and shaken for another 30 min at 37°C.

About 50 mg of feces was spiked with 0.5 mL water and homogenized for 5 min, and the mixtures were centrifuged at 13,000 rpm for 10 min to obtain 0.4 mL water supernatant. In addition, 0.5 mL methanol was added to the residue and homogenized once again. Next, 0.4 mL methanol supernatant was spiked with the water supernatant. After vortexing of the supernatant from the above steps for 10 s, 0.4 mL of fecal extract was transferred into a new tube with 10 μL of 1,2-^13^C-myristic acid (400 μg/mL), and then the samples were dried. A slight difference was exhibited for fecal samples when compared to serum samples in terms of derivatization. The volume of both methoxyamine hydrochloride pyridine solution and BSTFA was 60 μL, and the mixture was shaken for 60 min after adding BSTFA.

After derivatization, the mixture was centrifuged at 18,000 rpm for 10 min before injecting into the GC-MS. All derivatization must be performed in a waterless environment. Quality control (QC) samples of serum and feces were also prepared and derivatized according to the above protocol, and they were run after every 10 samples to monitor retention time and elution order of metabolites.

### GC-MS conditions for metabolomics analysis

The metabolomics analysis of serum and fecal samples was performed using the Trace 1310-TSQ 8000 Evo (Thermo Fisher, San Jose, CA, USA). The analytes were separated on a TG-5MS capillary column (0.25 mm × 30 m × 0.25 μm, Thermo Fisher, San Jose, CA, USA) with a split ratio of 20:1. The injection volume was 1 μL. The gradient heating program was conducted as follows: 0–1 min, 60°C; 1–14 min, 60–320°C; 14–19 min, 320°C. Helium (99.999%) was used as the carrier gas, with a flow rate of 1.2 mL/min. The TSQ 8000 was equipped with an Electron Ionization (EI) source. The ionization energy was 70 eV, the source temperature was 280°C, and the transfer line was held at 290°C. The GC-MS data were acquired after a solvent delay of 3.65 min, and the MS scan range was 50–500 m/z.

QC was assured by (a) randomization of sequence, (b) injection of QC samples between every 10 actual samples, and (c) checking the peak shape and intensity of the spiked internal standard (1,2-^13^C-myristic acid).

### Quantification of short-chain fatty acids (SCFAs) in rat feces

The SCFAs including acetate, propionate, butyrate, isobutyrate, valerate, and isovalerate were bacterial metabolites produced by the fermentation of dietary fiber and resistant starches with the effect of specific colonic anaerobic bacteria (Tan et al., 2014). SCFAs (butyrate in particular) are the major and preferred metabolic substrate for colonocytes, providing energy and playing a key role in promoting the integrity of the colon (Suzuki et al., 2008). Therefore, different concentrations of SCFAs could indicate changes in intestinal function. The fecal samples at day 36 were selected and extracted to determine the content of SCFAs. The detailed extraction protocol and GC-MS conditions are described in the Supplementary File.

### 16S rRNA-assisted high-throughput sequencing analysis

According to the fecal metabolomics and SCFAs results, RA might lead to alterations in the gut microbiota. Determination of gut microbiota differences between the control and model groups and whether ZT could normalize the changes in microbiota were critical aspects that needed to be explored. Thus, fecal samples from different groups were collected on day 36 and used for 16S rRNA gene analysis, including extraction of genome DNA, amplicon generation, PCR products quantification and qualification, PCR products mixing and purification, and library preparation and sequencing.

Total DNA from samples was extracted using the E.Z.N.A.Stool DNA kit, and the DNA concentration and purity were monitored on 1% agarose gels. Briefly, the V4–V5 domain of the 16S rRNA gene was amplified using primers 515F (5′-GTGCCAGCMGCCGCGG-3′) and 907R (5′-CCGTCAATTCMTTTRAGTTT-3′). PCR amplification was carried out in 30 μL reaction system containing 15 μL of Phusion®High-Fidelity PCR Master Mix (New England Biolabs), 0.2 μM of forward and reverse primers, and about 10 ng template DNA. Thermal cycling consisted of initial denaturation at 98°C for 1 min followed by 30 cycles of denaturation at 98°C for 10 s, annealing at 50°C for 30 s, elongation at 72°C for 60 s, and then a single final extension step at 72°C for 5 min. Then, mixed PCR products were purified with the GeneJET Gel Extraction Kit (Thermo Scientific). At last, the library was sequenced on an Illumina MiSeq platform, and 250 bp paired-end reads were generated. Finally, a total of 1,376,064 reads were obtained for subsequent analysis.

### Data processing and statistical analysis

GC-MS data (raw data files) of all samples were converted to ABF format using the ABF Converter (http://www.reifycs.com/AbfConverter/; Cajka et al., 2017). All the data were imported into the MS-DIAL (v.2.7.2) software program (Tsugawa et al., 2015) for peak detection, identification, and alignment using the following parameters: retention time beginning, 3.65 min; retention time end, 19 min; mass range beginning, 50 Da; mass range end, 500 Da; smoothing level, 3 scan; average peak width, 10 scan; retention index tolerance, 100,000; m/z tolerance, 0.5 Da; retention time tolerance for alignment, 0.3 min. Metabolite identification was performed by comparing MS spectra and Ankle RI index with those of the National Institute of Standards and Technology (NIST), and the metabolites were confirmed only when the EI similarity was above 80%. The alignment results were exported to a txt file labeled with the sample name, metabolites, peak height, and other information including retention time and quantification mass. Normalization is an important and effective method to exclude or reduce the unwanted overall variations in spectral data (Xi et al., 2014). In our research, data were normalized using sum normalization with MetaboAnalyst 4.0 (http://www.metaboanalyst.ca/) before statistical analysis. To search for changed metabolites in RA, data of normal distribution were obtained by log transformation. ANOVA and *q*-test were applied to search for differential metabolites. The metabolites were considered as potential altered metabolites according to the threshold of fold change >1.2 as well as *P* < 0.05.

## Results

### Quality evaluation of ZT by UHPLC-Q exactive-orbitrap-MS

**Figure 1:**
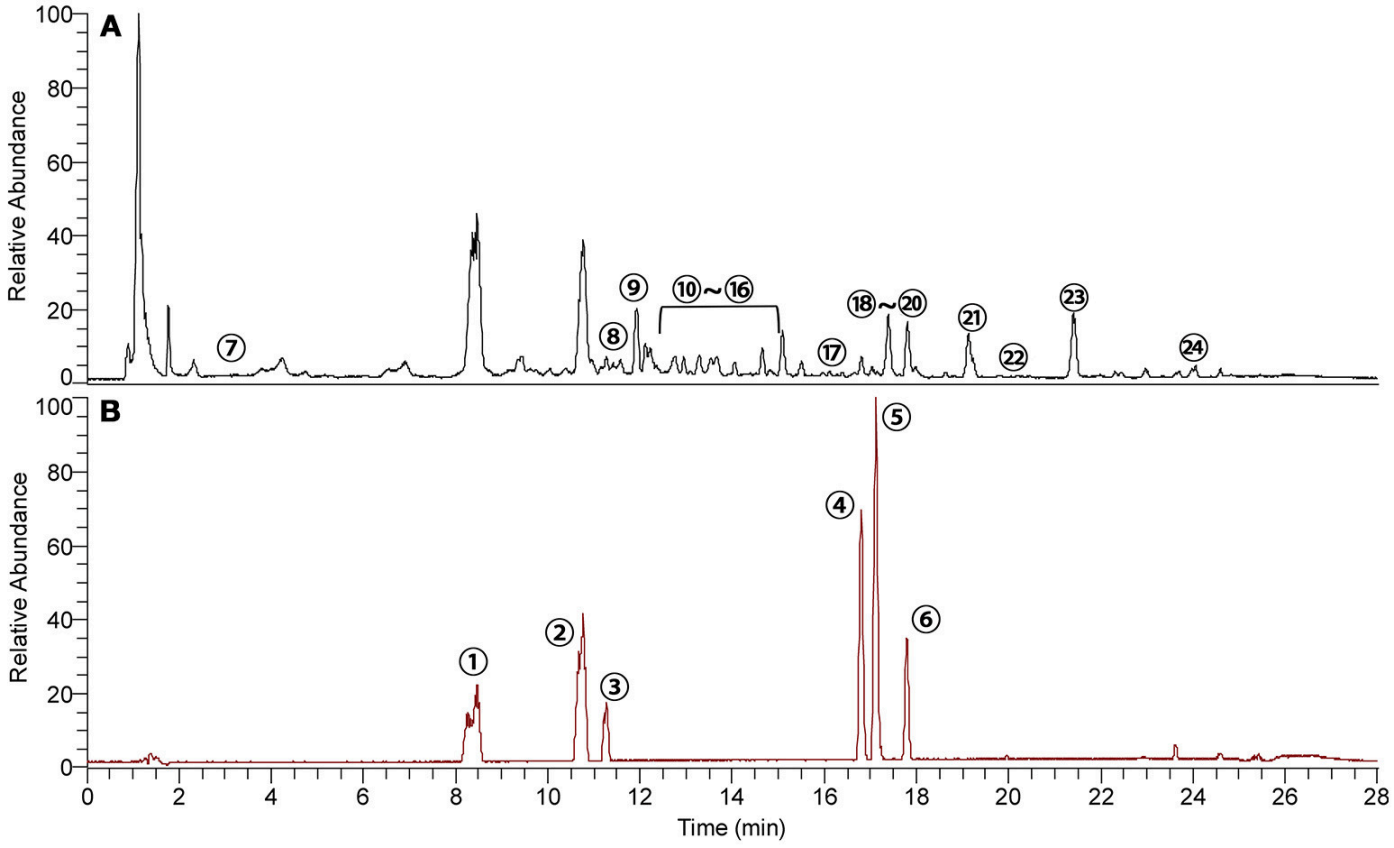
Typical base peak intensity chromatograms of **(A)** ZT extract and **(B)** a six-standard mixture analyzed by UHPLC-Q Exactive-Orbitrap-MS. The components are listed in Table 1.

The components in ZT were extracted and analyzed by UHPLC-Q Exactive-Orbitrap-MS and the total ion chromatogram (TIC) of ZT and six standards is shown in Figures 1A,B, respectively. Another 18 components in ZT were identified, and the results are summarized in Table 1. In addition, daphnetin, 7-hydroxycoumarin, and daphnoretin, three active ingredients that were reported to have therapeutic effects against RA (Shan et al., 2018), were determined at 1223.8, 485.1, and 131.3 μg in each tablet, respectively.

**Table 1:** The chemical components identified in the Zushima tablet.

**No**.	**Compound**	**RT(min)**	**m/z**	**Formula**	**Delta ppm**	**MS2**
1	Daphnetin	8.45	177.01845	C9H5O4	0.275	121.03
2	7-Hydroxycoumarin	10.77	161.02341	C9H5O3	0.179	133.03
3	Luteoloside	11.27	447.09354	C21H19O11	1.932	285.04
4	Apigenin	16.78	269.04556	C15H9O5	1.05	117.03
5	Kaempferol	17.11	285.04037	C15H9O6	1.196	
6	Daphnoretin	17.77	351.05011	C19H11O7	0.181	163.00, 191.00, 336.03
7	Daphnetin-8-O-β-D-glucopyranosyl-(1-6)-β-D-glucopyranoside	3.76	501.1246	C21H25O14	0.718	176.94
8	Daphjamilin	11.24, 12.72	467.09799	C24H19O10	0.717	277.05, 321.04
9	Giarldoid B	11.91	515.08264	C24H19O13	0.623	177.02, 339.07
10	Yuankanin	12.28	577.15619	C27H29O14	1.008	268.04, 283.06
11	(-)-pinoresinol-4-O-β-D-glucopyranoside	13.11	519.18549	C26H31O11	−0.598	83.01, 357.03
12	Daphgilin	13.53	353.02969	C18H9O8	0.496	177.01855
13	genkwanin-5-O-β-D-glucoside	13.03	445.11377	C22H21O10	0.847	268.04, 283.06
14	vladinolD	13.27	373.12851	C20H21O7	0.331	298.08, 313.11
15	Rutarensin	14.67	657.1449	C31H29O16	−0.111	351.08, 393.16
16	Luteolin	15.09	285.04074	C15H9O6	1.376	133.03
17	Daphnodorin A/C/D1/D2	16.11/16.39/18.00	525.11841	C30H21O9	0.401	119.05,151.00, 431.08
18	Daphneticin	16.29	385.09341	C20H17O8	0.276	175.93,209.09, 121.03
19	(-)-pinoresinol	17.05	357.13474	C20H21O6	0.465	83.01, 122.04,137.06, 221.08
20	(-)-Dihydrosesamin	17.18	355.1192	C20H19O6	0.455	160.05, 176.01, 219.07
21	Daphneolone	19.11	269.11874	C17H17O3	0.549	93.03, 135.04
22	12-hydroxydaphnetoxin	20.21	497.18298	C27H29O9	0.541	121.03, 327.12
23	Genkwanin	21.41	283.06119	C16H11O5	1.06	268.04
24	Daphnetoxin	23.91	481.18756	C27H29O8	0.986	121.03

### Basic physiological parameters

**Figure 2:**
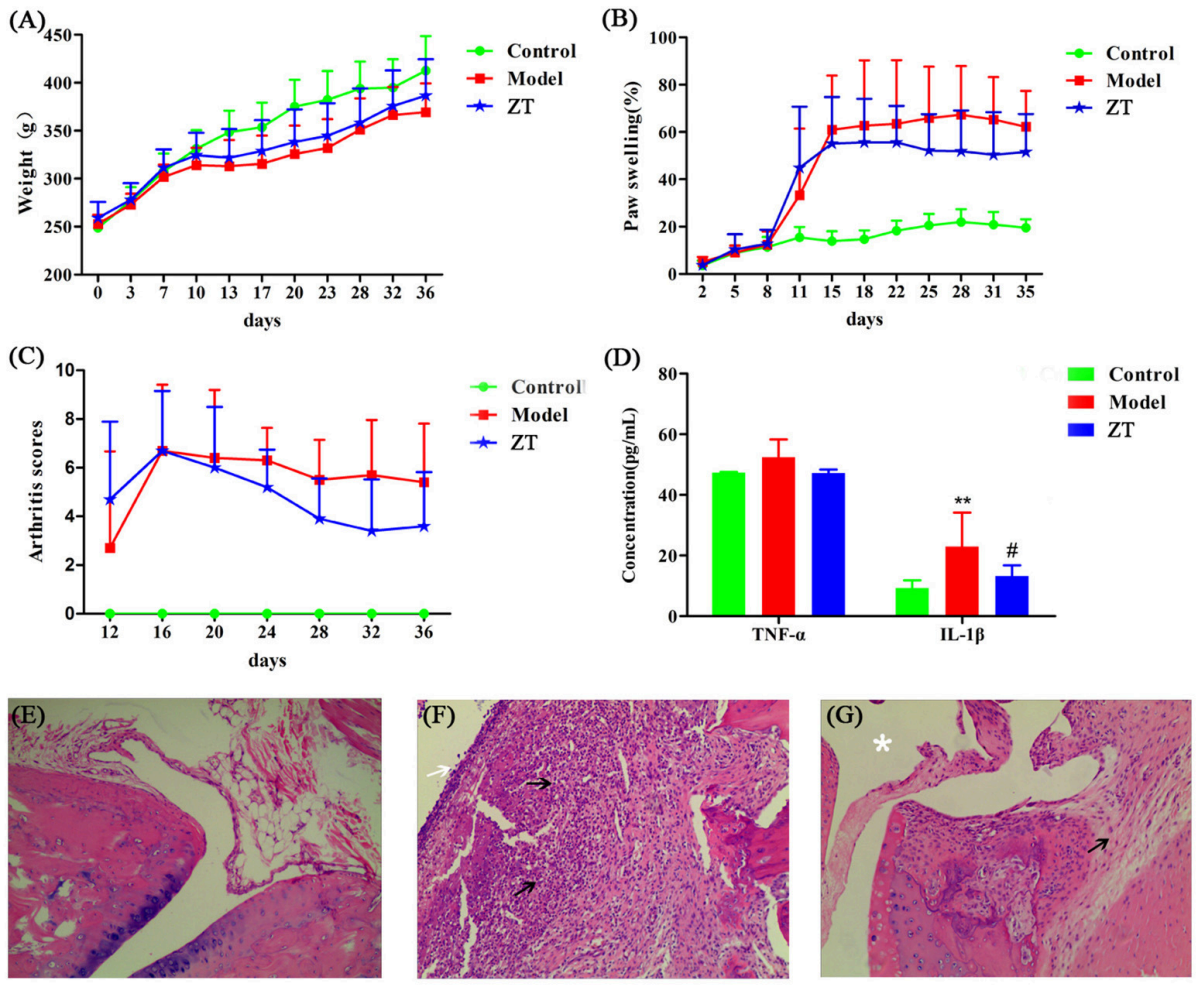
Evaluation of CIA model construction and efficacy of ZT. **(A)** Body weight, **(B)** paw swelling, **(C)** arthritis score, and **(D)** serum levels of inflammatory factors TNF-α and IL-1β. All the above values are expressed as Mean±SD (*n* = 10), ^**^, vs. Control, *P* < 0.01; #, vs. Model, *P* < 0.05. Representative photographs of histological examination of synovial tissues in the ankle joint (100 × magnification). **(E)** Control group, **(F)** model group, **(G)** ZT group. The black arrows indicate numbers of inflammatory cells, such as plasma cells, lymphocytes, and neutrophils, the white arrow indicate destoryed cartilage. The asterisks indicate clear joint cavity.

Most of the rats fell ill on day 11 after primary immunization and exhibited hind limb stiffness, reduced activity, and ankle and toe inflammation. Four of the twenty-four rats failed to develop the disease and the incidence of RA was up to 83.33%. Body weight, foot swelling, and arthritis scores are shown in Figures 2A–C. During the experiments, the body weight of rats in the model and ZT groups increased slowly compared to that in the control group. The percentage of foot swelling in CIA rats increased rapidly on day 11, and the swelling was attenuated by the oral administration of ZT. Arthritis scores also indicated that ZT demonstrated beneficial effects in CIA rats.

### Quantification of serum TNF-α and IL-1β and histopathology analysis

Our study showed that serum IL-1β level was higher in the model group than in the control and ZT groups, while TNF-α did not exhibit any obvious difference among the three groups (Figure 2D). After hematoxylin-eosin (HE) staining, the synovial tissues of the ankle joint were observed under a microscope (Figures 2E–G). CIA rats exhibited obvious distinct synovial hyperplasia, and increased numbers of inflammatory cells, such as plasma cells, lymphocytes, and neutrophils, were present in the synovium. In the ZT group, inflammation was markedly attenuated and only few inflammatory cells were observed. In summary, the above results indicated that ZT could significantly inhibit inflammatory responses.

### System stability and reproducibility analysis

Both serum and fecal samples were analyzed based on GC-MS to obtain biochemical information on metabolites. The GC-MS TICs of serum and feces are shown in Figures S1A,B. To evaluate system stability and reproducibility, PCA analysis was performed to process the data matrix of QC samples. As shown in Figures S2A,B, in PCA score plots of serum and fecal samples, QC samples were clustered, which indicated that the stability of the GC-MS system was good throughout the whole analysis. In addition, the relative standard deviations (RSDs) of the peak height of 1, 2-^13^C-myristic acid was 15.39% for serum and 15.00% for feces, indicating that the analytical conditions and sample process exhibited good repeatability and stability for the metabolomics study.

### Differential metabolites in serum and fecal samples

In serum samples, 95 endogenous metabolites were identified, including amino acids, glucose, and long-chain fatty acids. In addition, 99 endogenous metabolites were detected in fecal samples, including amino acids, amides, and pyrimidines. Fifty-one metabolites were detected in both the serum and feces, and these metabolites contained pyruvic acid, lactic acid, urea, and some amino acids.

**Figure 3:**
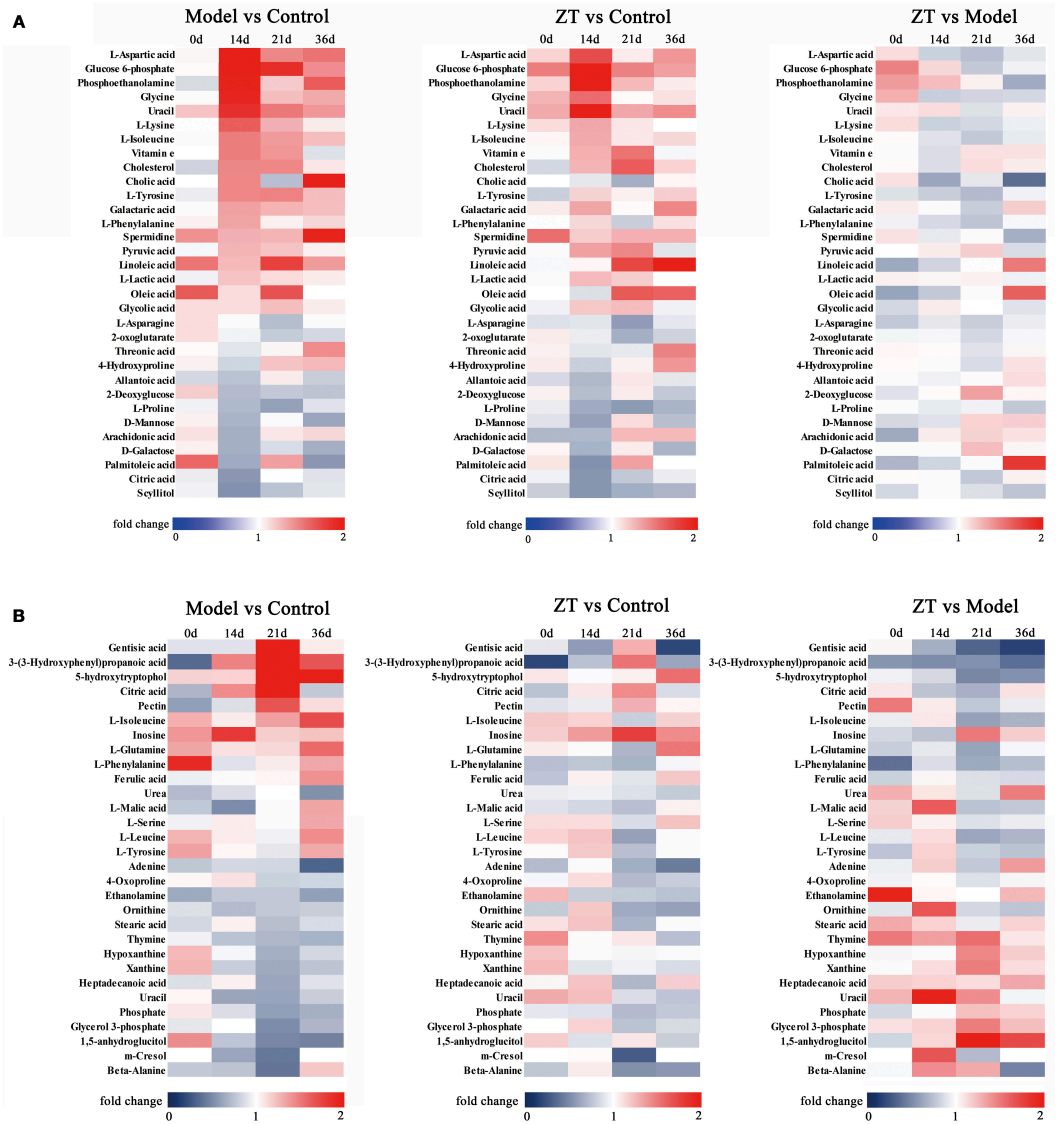
Heatmap of identified differential metabolites with serum **(A)** and fecal **(B)** metabolomics profile. Each cell in the heatmap represents the fold change of a particular metabolite.

The differential metabolites were selected based on one-way ANOVA and q test (*P* < 0.05), and there was a fold change >1.2 between the control and model groups on the same day. We filtered 31 potential biomarkers in the serum and 30 in the feces by comparing the model group with the control group. The detailed information about altered metabolites in the serum and fecal samples is presented in Tables 2, 3, respectively. To illustrate the fluctuations in altered serum and fecal metabolites and the content difference among the three groups, metabolic heatmaps of serum and feces were also generated (Figures 3A,B).

**Table 2:** Significant serum metabolites associated with rheumatoid arthritis based on gas chromatography-mass spectrometry.

**No**.	**Match**	**RT(min)**	**Quant mass (m/z)**	**Day**	**ANOVA**	***q*-test**
1	Allantoic acid	10.05, 10.93	331, 259	14	0.011^b^	0.027^b^
2	Scyllitol	10.45	318	14	<0.001^b^	<0.001^b^
3	Vitamin e	15.29	237	21	0.015^c^	0.021^c^
4	Arachidonic acid	12.21	73	14	0.031^b^	0.012^b^
5	L-Asparagine	7.79, 8.38, 8.81	84, 116, 73	21	<0.001^c^	0.013^c^
6	L-Aspartic acid^*^	7.91, 7.28	232, 73	14	<0.001^b^	<0.001^b^
7	Cholesterol	15.46	129	21	0.003^c^	0.006^c^
8	Cholic acid	16.28	253	36	0.001^d^	0.002^d^
9	Citric acid^*^	9.67	273	14	<0.001^b^	<0.001^b^
10	D-Galactose	10.15	73	14	0.027^b^	0.015^b^
11	Glucose 6-phosphate	12.15	299	14	0.01^b^	0.018^b^
12	Glycine	5.07, 6.51	102, 174	14	0.008^b^	0.002^b^
13	Glycolic acid	4.73	147	21	0.014^c^	0.004^c^
14	L-Isoleucine^*^	6.41	158	14,21	0.003^b^, 0.006^c^	0.001^b^, 0.029^c^
15	L-Lactic acid	4.61	207	14	0.023^b^	0.028^b^
16	Linoleic acid^*^	11.51	75	0,21	0.04^a^, <0.001^c^	0.03^a^, <0.001^c^
17	L-Lysine^*^	10.19	174	14	0.037^b^	0.011^b^
18	D-Mannose	10.12	87	14,36	0.008^b^, 0.034^d^	0.015b, 0.01^d^
19	Galactaric acid	10.69	333	14	0.009^b^	0.006^b^
20	Oleic acid	11.53	339	0, 21	0.013^a^, 0.006^c^	0.008^a^, 0.008^c^
21	O-Phosphoethanolamine	9.48	299	14	0.012^b^	0.025^b^
22	Palmitoleic acid	10.63	311	0,14,36	0.04^a^, 0.014^b^, 0.003^d^	0.017^a^, 0.011^b^, 0.002^d^
23	L-Phenylalanine^*^	8.11, 8.59	120, 73	14	0.004^b^	0.001^b^
24	L-Proline^*^	6.45	142	14, 21	0.006^b^, 0.014^c^	0.017^b^, 0.016^c^
25	Pyruvic acid	4.58	174	21	0.021^c^	0.04^c^
26	Spermidine	11.8	144	36	<0.001^d^	<0.001^d^
27	Threonic acid	8.19	73	36	0.014^d^	0.011^d^
28	4-Hydroxyproline	9.41, 7.96	274, 230	14	0.045^b^	0.045^b^
29	L-Tyrosine	10.01, 10.28	179, 218	14	0.011^b^	0.003^b^
30	Uracil	6.72	241	14,21	<0.001^b^, 0.005^c^	<0.001^b^, 0.001^c^
31	2-Deoxyglucose	9.29	205	14	0.036^b^	0.028^b^

a 0 day;

b 14 days;

c 21 days;

d 36 days;

* *confirmed with pure standards*.

**Table 3:** Significant fecal metabolites associated with rheumatoid arthritis based on gas chromatography-mass spectrometry.

**No**.	**Biomarkers**	**RT(min)**	**Quant mass (m/z)**	**Day**	**ANOVA**	***q*-test**
1	1,5-anhydroglucitol	9.84	73	21	0.007^c^	0.012^c^
2	3-(3-Hydroxyphenyl)-propanoic acid	9.29	310	21	0.043^c^	0.02^c^
3	4-Oxoproline	7.95	156	21	0.008^c^	0.046^c^
4	5-hydroxy-3-indoleacetic acid	11.49	290	21	0.033^c^	0.026^c^
5	Adenine	9.93	264	36	0.013^d^	0.006^d^
6	Beta-Alanine	7.32	248	21	0.029^c^	0.009^c^
7	Citric acid^*^	9.66	273	21	0.003^c^	0.001^c^
8	Ethanolamine	6.22	174	36	0.004^d^	0.001^d^
9	Gentisic acid	9.4	355	21	0.001^c^	0.001^c^
10	Glycerol 3-phosphate	9.35	299	21	0.004^c^	0.001^c^
11	Heptadecanoic acid	11.63	315	21	0.019^c^	0.007^c^
12	Hypoxanthine	9.59	265	21	0.005^c^	0.003^c^
13	Inosine	13.09	281	14	0.049^b^	0.018^b^
14	L-Glutamine	9.39	73	36	0.007^d^	0.005^d^
15	L-Isoleucine^*^	6.41	158	36	0.004^d^	0.001^d^
16	L-Leucine^*^	6.25	158	36	0.006^d^	0.005^d^
17	L-Malic acid	7.7	73	14	0.041^b^	0.013^b^
18	L-Phenylalanine^*^	8.11, 8.59	120, 73	36	0.025^d^	0.026^d^
19	L-Serine^*^	6.13, 6.87	73, 73	36	0.008^d^	0.003^d^
20	L-Tyrosine	10.28	280	36	0.005^d^	0.005^d^
21	m-Cresol	5.34	165	21	<0.001^c^	0.005^c^
22	Ornithine	8.48, 8.9, 9.65	142, 328, 73	21	0.002^c^	0.024^c^
23	Pectin	8.8	73	21	0.022^c^	0.006^c^
24	Phosphate	6.26	299	21	0.004^c^	0.001^c^
25	Stearic acid	11.62	341	21	0.013^c^	0.02^c^
26	Thymine	7.15	255	36	0.034^d^	0.013^d^
27	Ferulic acid	11	323	36	0.043^d^	0.015^d^
28	Uracil	6.72	241	21	0.019^c^	0.008^c^
29	Urea	5.94	147	0,36	0.011^a^, 0.037^d^	0.004^a^, 0.006^d^
30	Xanthine	10.69	353	21	0.013^c^	0.007^c^

a 0 day;

b 14 days;

c 21 day;

d 36 days;

* *confirmed with pure standards*.

### Metabolic pathway analysis

**Figure 4:**
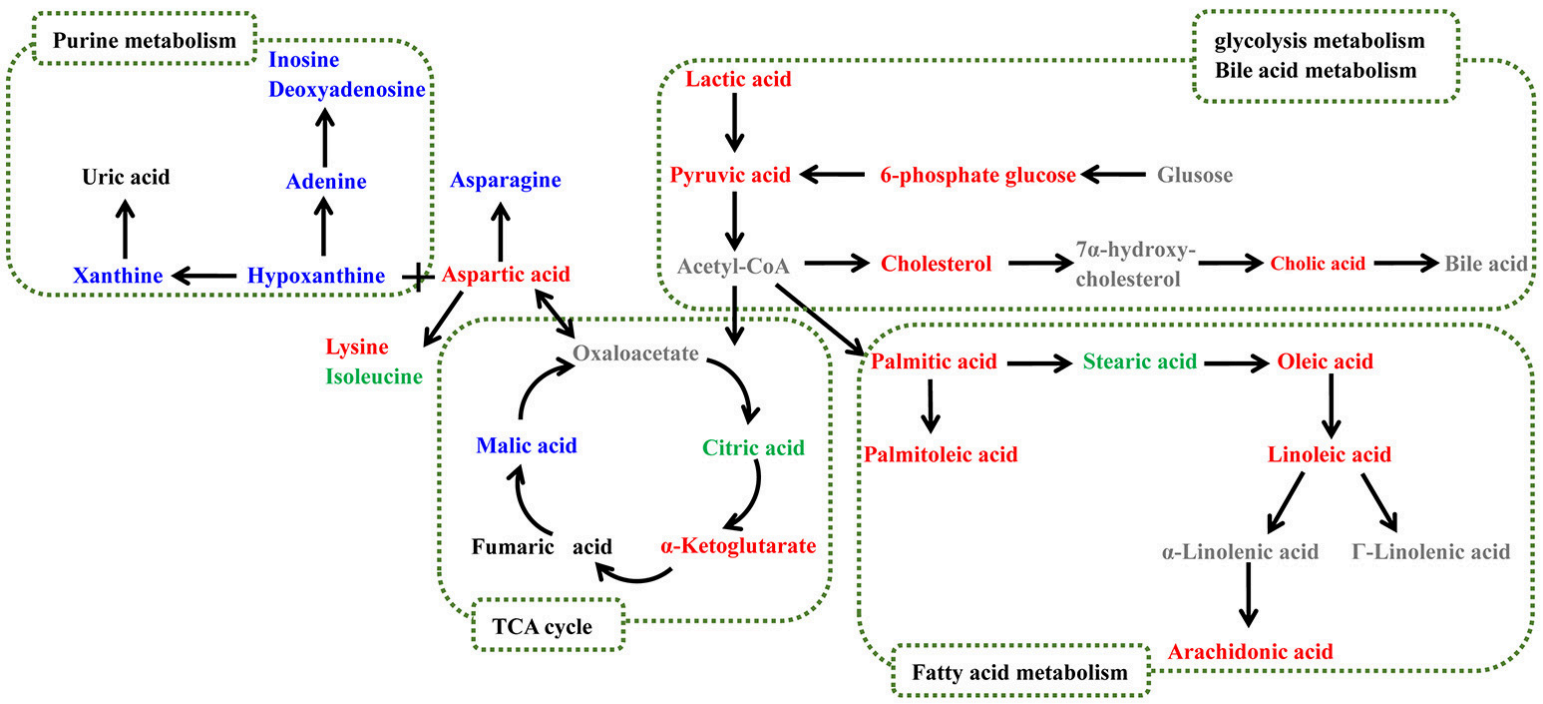
Potential metabolic pathways disturbed in CIA rats. Metabolite names denoted in red or blue indicate that they were found in the serum or feces, respectively, and the metabolites shown in green were found in both. The names of the possible metabolic pathways are denoted in the green dotted box.

Biological pathway analysis was performed based on MetaboAnalyst 4.0, Kyoto Encyclopedia of Genes and Genomes (KEGG) (http://www.kegg.jp/), and Human Metabolome Database (HMDB) (http://www.hmdb.ca/). The main biochemical pathways related to RA included the TCA cycle, glycolysis metabolism, fatty acid metabolism, and purine metabolism. The metabolic pathway map is shown in Figure 4.

### Quantification of SCFAs in fecal samples

**Figure 5:**
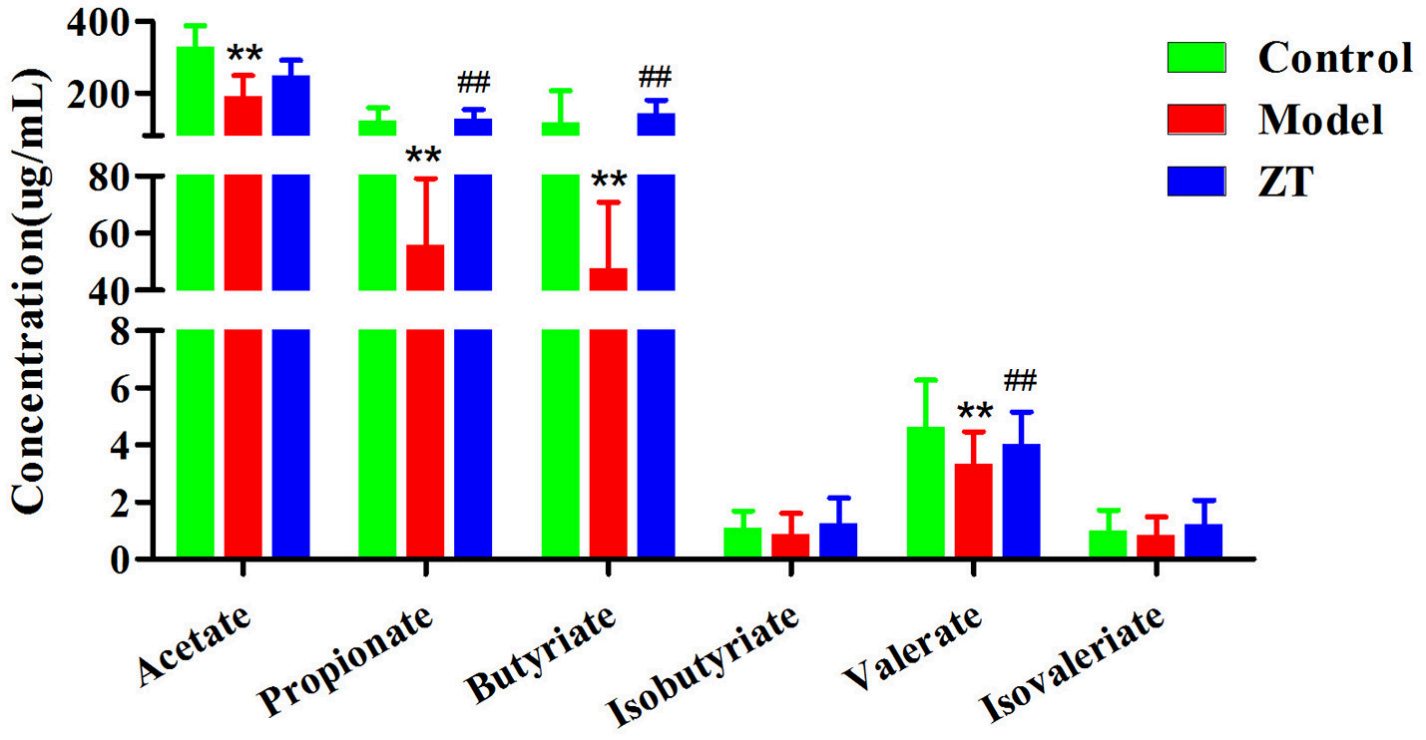
The concentration of SCFAs in rat fecal samples in control, CIA rats, and ZT-treated rats. ^**^, vs. Control, *P* < 0.01; ##, vs. Model, *P* < 0.01.

As shown in Figure 5, acetate, propionate, and butyrate were the three most abundant SCFAs in rat feces. Compared with the control, the concentrations of all SCFAs decreased in CIA rats, especially acetate, propionate, butyrate, and valerate (*P* < 0.01), which indicated that the gut microbiota was disturbed under the influence of RA. The results also showed that ZT could regulate the content of propionate, butyrate, and valerate in the feces.

### Diversity analysis of gut microbiota in fecal samples

**Figure 6:**
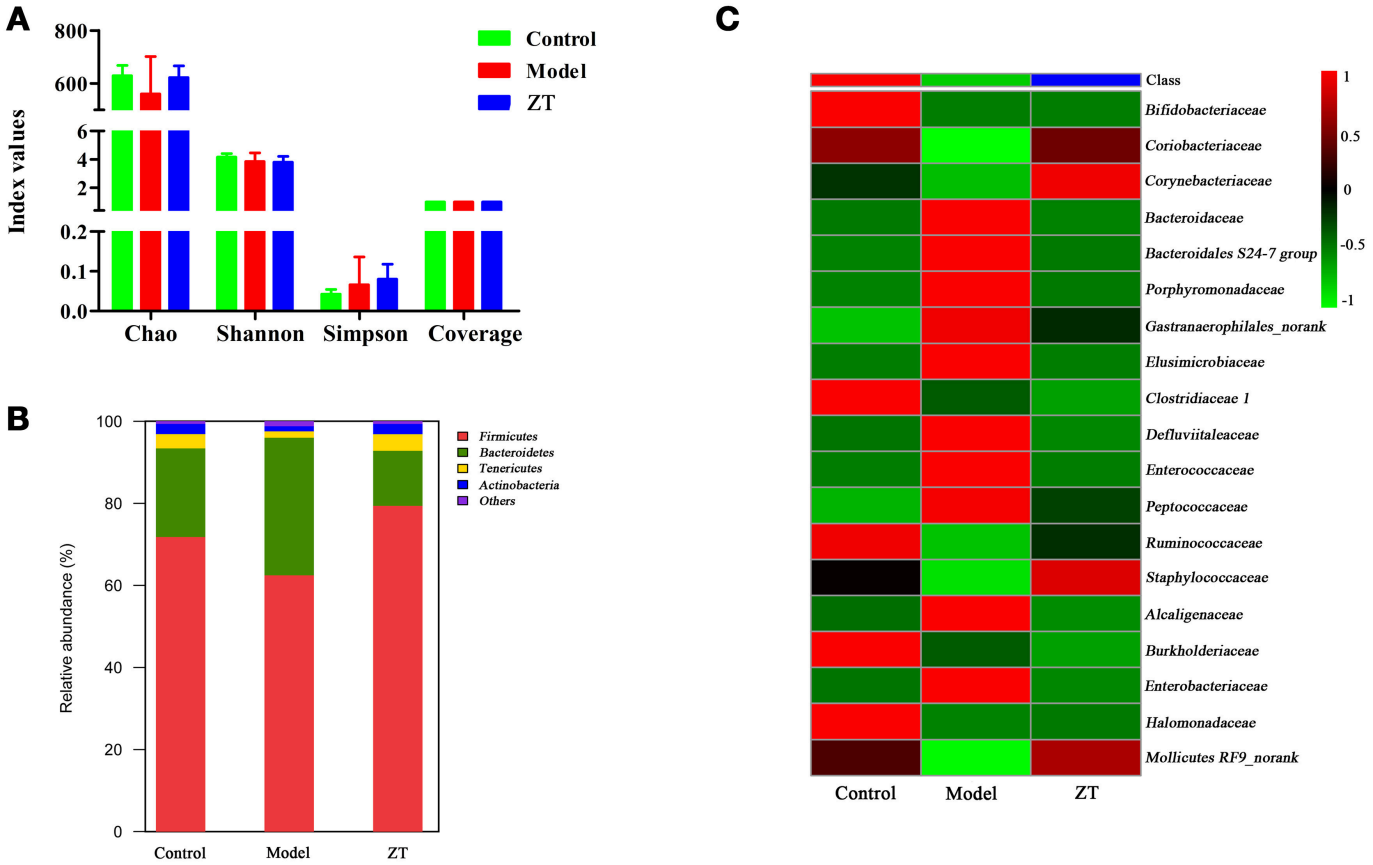
The results of 16S rRNA sequence analysis. **(A)** α-diversity of chao, shannon, simpson, and coverage indices in control, CIA rats, and ZT-treated rats. **(B)** Gut microbiome composition profiles at the phylum level in control, CIA rats, and ZT-treated rats. **(C)** Microbial community of altered bacteria at the family level in control, CIA rats, and ZT-treated rats.

Based on fecal metabolomics and SCFA results, we speculated that RA might cause gut microbiota disorder. Therefore, gut microbiota diversity analysis was further performed to confirm the changes in gut microbiota and to discover the bacteria responsible for the altered metabolic profile of fecal samples. Diversity analysis, including α-diversity and community structure, was performed based on the UPARSE software package. α-diversity usually reflects the abundance of gut microbiota, and the microbial community structure is used to observe the community structure in different groups at the taxonomic level (phylum, family, and genus). Figure 6A shows four indices: Chao, Shannon, Simpson, and Coverage in different groups. The results indicate that α-diversities of the gut microbiome in different groups did not show significant differences. Figure 6B shows the identified gut microbiota assigned at the phylum level, with each color representing an individual bacterial phylum. The results showed that *Firmicutes* (62.5–79.4%) and *Bacteroidetes* (13.4–21.6%) were the primary bacteria in the gut microbiota of SD rats, followed by *Tenericutes* (1.6–3.5%) and *Actinobacteria* (1.3–2.5%), while only *Actinobacteria* exhibited an obvious difference between the control and model groups (*P* < 0.05). Therefore, we decided to explore the difference at the family level between control and RA rats. Finally, we found that the content of 19 bacteria at the family level changed in model rats compared with controls, and the heatmap of these bacteria among three groups is shown in Figure 6C. In general, most of these changed bacteria belonged to the *Actinobacteria, Bacteroidetes, Firmicutes*, and *Proteobacteria* phylum while ZT showed a regulatory effect on *Coriobacteriaceae, Bacteroidaceae, Porphyromonadaceae*, among others.

## Discussion

### Pathway analysis of serum metabolomics

The identified serum biomarkers included l-phenylalanine, l-aspartic acid, citric acid, glucose 6-phosphate, cholic acid, and cholesterol. It has been reported that these metabolites are related to the TCA cycle, glycolysis metabolism, and fatty acid metabolism.

### TCA cycle and glycolysis metabolism

The TCA cycle, which takes place in the mitochondria, is a key link in the metabolism of carbohydrates, lipids, and amino acids. The intermediates of the TCA cycle are also precursors of many biosynthetic pathways (Deng et al., 2016). The rate of the TCA cycle reflects the condition of energy metabolism. In this paper, the levels of citric acid decreased in the model group compared with the control group, indicating insufficient energy supply. In addition, it was reported that systemic inflammation induced by RA could lead to an increase in energy metabolism (Zhou et al., 2016). Within our identified altered metabolites, the level of l-isoleucine, an essential amino acid that can supply energy, was increased in CIA rats. When the rats were orally administered ZT, the serum level of l-isoleucine decreased, indicating that ZT was able to modulate energy metabolism.

Another metabolic pathway related to energy metabolism is glycolysis metabolism, which plays a key role in supplying ATP. In our study, the levels of pyruvate, lactic acid, and glucose 6-phosphate markedly increased during disease progression, indicating that the activity of glycolysis was enhanced.

Pyruvate could stimulate the transcription of vascular endothelial growth factor (VEGF) mRNA (Lee et al., 2001). In our previous study (Zhang et al., 2014), we found that the level of VEGF in RA synovial tissue increased significantly, and ZT decreased these levels, indicating that ZT could reduce the symptoms of RA by modulating the content of pyruvate. In addition, degradation of proline, which is associated with the TCA and urea cycle, was proven to fulfill the demand of ATP (Pandhare et al., 2009).

Smolenska et al. (2016) investigated the plasma concentrations of amino acid and nicotinamide metabolites in patients with RA and found that the level of l-aspartic acid and l-phenylalanine increased, while that of l-asparagine decreased in patients with RA. These results were confirmed in our experiments. Moreover, ZT markedly modulated the level of l-aspartic acid and l-phenylalanine. RA was reported to cause dysfunction of protein synthesis and affect amino acid metabolism (Shin et al., 2011).

### Fatty acid metabolism

Another pathway related to RA is fatty acid metabolism. Among the differential metabolites in serum samples, palmitoleic acid, oleic acid, linoleic acid, and arachidonic acid were reported to participate in fatty acid metabolism. Fatty acids could also supply energy and serve as important sources of various lipid species (Van Meer et al., 2008). Unsaturated fatty acids can promote the production of inflammatory cytokines TNF-α and IL-1β, and then increase the production of PGE2 and NO, the two important inflammatory factors related to RA (Bruusgaard and Andersen, 1975; Ralston and Grabowski, 1996). In our investigation, ZT regulated the content level of palmitoleic acid markedly.

In accordance with our previous findings (Peng et al., 2017), markedly upregulated metabolites (linoleic acid and oleic acid) in fatty acid metabolism were observed in this investigation. The levels of both cholesterol and cholic acid were higher in RA rats than in the controls. The accumulation of cholesterol could promote inflammatory responses, inflammasome activation, and the production of monocytes as well as neutrophils.

### Pathway analysis of fecal metabolomics

The metabolomics study of feces was implemented to observe the altered metabolic profiles of gut microbiota and host co-metabolites. Fecal metabolomics presented different results from those of the serum. As shown in Table 3, our identified biomarkers included l-tyrosine, m-cresol, and nucleotide metabolites such as uracil, hypoxanthine, and thymine, which have been reported as gut microbiota-related metabolites (Zheng et al., 2013). The results suggested that the gut microbiota play an important role in RA-induced metabolic changes. According to the fecal metabolomics results, the important pathway related to RA was purine metabolism.

### Purine metabolism

It was reported that the level of uric acid increased in patients with RA, and excessive uric acid was deposited in the joints, soft tissues, and cartilage (Khondker and Khan, 2014; Liu et al., 2016). In our experiments, uric acid and its downstream products hypoxanthine, xanthine, and inosine were all detected, and they were all listed as biomarkers in fecal samples, indicating that RA had markedly affected purine metabolism. Inosine is an intermediate in the degradation of purines, while hypoxanthine is a naturally occurring purine derivative and a reaction intermediate in adenosine metabolism.

As reported previously (Nakajima et al., 2014), reactive oxygen species (ROS) were involved in the pathobiology of RA, and uric acid, the final product of purine and pyrimidine, could get eliminate ROS. Oxidative stress has also been implicated in the pathogenesis of RA (Charles-Schoeman et al., 2017). In addition, it was reported that daphnetin in ZT could reduce lipopolysaccharide (LPS)-induced production of ROS, indicating that ZT would exhibit anti-inflammatory effects probably by regulating the level of purine (Shen et al., 2017).

### The role of SCFAs in collagen-induced arthritis (CIA)

It was reported that the severity of type II CIA was decreased after oral administration of either acetate, propionate, or butyrate (Mizuno et al., 2017), which indicated the significant role of SCFAs in the regulation of inflammation. This conclusion could also be confirmed in our experiment because the concentrations of propionate, butyrate, and valerate increased in the ZT group. A possible reason for this result maybe that SCFAs could inhibit the disease by increasing the number of Tregs, a T cell subset for maintaining the immune response and suppressing excessive immune response (Zhou et al., 2015).

### Correlation between gut microbiota and metabolites of serum and feces

It was reported that RA is a typical complex polygenic disease mediated by the human leukocyte antigen (HLA) system, and genetic differences largely determine the human body's susceptibility to the disease (Cai et al., 2012). According to the US WebMD Medical News (Taneja, 2014), the link between the genetic predisposition to RA and environmental factors may depend on the gut microbiota. Several studies supported this point of view and indicated that the gut microbiota is crucial to maintain the balance of the immune system in patients with RA. Gomez et al. explored the interaction between genetic factors and gut microbiota, and demonstrated that altered gut microbiota could predict the body's susceptibility to RA (Gomez et al., 2012). *Lactobacillus* could significantly reduce serum TNF-α, IL-6, and other proinflammatory cytokines in patients with RA (Li et al., 2015a). In our present study, the correlation between changed gut microbiota and serum and fecal biomarkers and SCFAs was analyzed based on Pearson's correlation coefficient, and the *r*-values are presented in Tables 4–**6**, while the *P*-values are shown in Tables S2–S4. The gut microbiota is closely related to the bile acid pool. Primary bile acids were synthesized in the liver from cholesterol (Chiang, 2009). The gut microbiota modifies the bile acid steroid core and transforms the primary bile acids to secondary bile acids. Along with the change in the gut microbiome between control and CIA rats, the bile acid pool was also disturbed. As shown in Table 4, cholic acid was highly correlated with the gut microbiome, including *Bacteroidaceae, Bacteroidales S24-7 group, Enterococcaceae*, and *Corynebacteriaceae*.

**Table 4:** The *r*-value of correlation analysis for perturbed gut bacteria families and altered serum metabolites on day 36 (^*^P < 0.05; ^**^P < 0.01).

	**Cholic acid**	**D-mannose**	**Palmitoleic acid**	**Spermidine**	**Threonic acid**
*Bifidobacteriaceae*	−0.205	0.463^*^	0.208	−0.344	−0.491^**^
*Coriobacteriaceae*	−0.411^*^	0.446^*^	0.154	−0.538^**^	−0.155
*Corynebacteriaceae*	−0.511^**^	0.287	0.285	−0.466^**^	−0.079
*Bacteroidaceae*	0.565^**^	−0.6^**^	−0.317	0.392^*^	0.083
*Bacteroidales S24-7 group*	0.411^*^	−0.411^*^	−0.365^*^	0.257	−0.093
*Porphyromonadaceae*	0.447^*^	−0.593^**^	−0.38^*^	0.407^*^	0.073
*Gastranaerophilales_norank*	0.578^**^	−0.406^*^	−0.369^*^	0.422^*^	0.216
*Elusimicrobiaceae*	0.154	−0.134	−0.155	0.418^*^	−0.038
*Clostridiaceae 1*	−0.012	0.259	0.051	−0.243	−0.408^*^
*Defluviitaleaceae*	0.462^*^	−0.026	−0.526^**^	0.528^**^	0.274
*Enterococcaceae*	0.409^*^	−0.361	−0.468^**^	0.487^**^	0.021
*Peptococcaceae*	0.306	−0.164	−0.322	0.16	0.241
*Ruminococcaceae*	−0.481^**^	0.386^*^	0.158	−0.643^**^	−0.321
*Staphylococcaceae*	−0.519^**^	0.333	0.459^*^	−0.629^*^	−0.243
*Alcaligenaceae*	0.395^*^	−0.417^*^	−0.357	0.417^*^	0.214
*Burkholderiaceae*	0.004	0.147	0.059	−0.421^*^	−0.266
*Enterobacteriaceae*	0.515^**^	−0.261	−0.471^**^	0.586^**^	0.077
*Halomonadaceae*	−0.088	0.482^**^	0.346	−0.355	−0.385^*^
*Mollicutes RF9_norank*	−0.529^**^	0.303	0.178	−0.499^**^	−0.19

It was also observed (Table 5) that the *Defluviitaleaceae* family was positively and highly correlated with l-isoleucine, l-leucine, and l-phenylalanine while *Defluviitaleaceae* was reported to regulate the metabolism of amino acids, energy, and carbohydrates (Zhao et al., 2018). *Halomonadaceae* was negatively correlated with ferulic acid, l-glutamine, and l-isoleucine in our research.

**Table 5:** The *r*-value of correlation analysis of perturbed gut bacteria families and altered fecal metabolites on day 36 (^*^P < 0.05; ^**^P < 0.01).

	**Adenine**	**Ethanolamine**	**Ferulic acid**	**Glutamine**	**L-Isoleucine**	**L-Leucine**	**L-Phenylalanine**	**L-Serine**	**Thymine**	**L-Tyrosine**
*Bifidobacteriaceae*	0.393^*^	0.307	−0.372^*^	−0.311	−0.279	−0.166	−0.273	−0.381^*^	−0.017	−0.381^*^
*Coriobacteriaceae*	0.128	0.323	−0.419^*^	−0.151	−0.19	−0.188	−0.147	−0.084	0.267	−0.188
*Corynebacteriaceae*	0.245	0.324	−0.053	0.087	−0.09	−0.002	0.093	0.152	0.372	0.046
*Bacteroidaceae*	−0.143	−0.339	0.233	0.002	0.198	0.165	0.056	−0.05	−0.272	0.116
*Bacteroidales S24-7 group*	−0.209	−0.324	0.158	0.036	0.209	0.179	0.092	0.014	−0.188	0.122
*Porphyromonadaceae*	−0.127	−0.384^*^	0.317	0.047	0.287	0.245	0.141	0.076	−0.234	0.228
*Gastranaerophilales_norank*	−0.265	−0.475^**^	0.305	0.289	0.399^*^	0.278	0.111	0.068	−0.459^*^	0.12
*Elusimicrobiaceae*	−0.09	−0.452^*^	0.437^*^	0.072	0.021	0.107	0.017	0.105	−0.046	0.118
*Clostridiaceae 1*	0.1	0.088	−0.443^*^	−0.216	−0.068	0.015	0.052	−0.228	0.025	0.024
*Defluviitaleaceae*	−0.339	−0.33	0.123	0.282	0.489^*^	0.43^*^	0.376^*^	0.182	−0.357	0.312
*Enterococcaceae*	−0.149	−0.407^*^	0.274	0.099	0.257	0.304	0.262	0.154	−0.12	0.286
*Peptococcaceae*	−0.503^**^	−0.174	−0.017	0.21	0.259	0.134	0.056	0.035	−0.35	0.078
*Ruminococcaceae*	0.327	0.279	−0.531^**^	−0.319	−0.294	−0.265	−0.155	−0.24	0.104	−0.257
*Staphylococcaceae*	0.424^*^	0.521^**^	−0.183	0.003	−0.161	−0.055	0.013	0.002	0.374^*^	−0.081
*Alcaligenaceae*	−0.219	−0.573^**^	0.154	−0.067	0.216	0.167	0.213	0.072	−0.111	0.283
*Burkholderiaceae*	0.295	0.172	−0.394^*^	−0.216	−0.158	−0.072	−0.049	−0.132	0.132	−0.114
*Enterobacteriaceae*	−0.236	−0.414^*^	0.223	0.072	0.294	0.235	0.111	0.074	−0.241	0.158
*Halomonadaceae*	0.255	0.269	−0.535^**^	−0.403^*^	−0.401^*^	−0.292	−0.229	−0.36	0.264	−0.274
*Mollicutes RF9_norank*	0.215	0.316	−0.336	−0.107	−0.201	−0.164	−0.043	−0.095	0.111	−0.121

As shown in Table 6, acetate, propionate, butyrate, and valerate showed obvious correlation with almost all species, and the *Bacteroidales S24-7 group* in particular. The *Bacteroidales S24-7 group*, also named *Candidatus Homeothermaceae*, is capable of producing acetate, propionate, and succinate (Ormerod et al., 2016). However, in our research, it was negatively correlated with all SCFAs except isobutyrate and isovalerate, and further exploration is needed to determine the reason.

**Table 6:** The *r*-value of correlation analysis of perturbed gut bacteria families and short chain fatty acids on day 36 (^*^P < 0.05; ^**^P < 0.01).

	**Acetate**	**Propionate**	**Butyrate**	**Isobutyrate**	**Valerate**	**Isovalerate**
*Bifidobacteriaceae*	0.667^**^	0.352	0.144	0.1	0.333	0.161
*Coriobacteriaceae*	0.404^*^	0.556^**^	0.414^*^	0.344	0.411^*^	0.356
*Corynebacteriaceae*	0.229	0.264	0.446^*^	0.296	0.218	0.223
*Bacteroidaceae*	−0.332	−0.444^*^	−0.343	−0.231	−0.33	−0.25
*Bacteroidales S24-7 group*	−0.457^*^	−0.459^*^	−0.413^*^	−0.348	−0.477^**^	−0.304
*Porphyromonadaceae*	−0.373^*^	−0.403^*^	−0.348	−0.315	−0.412^*^	−0.335
*Gastranaerophilales_norank*	−0.45^*^	−0.373^*^	−0.207	0.014	−0.14	0.073
*Elusimicrobiaceae*	−0.075	−0.346	−0.391^*^	−0.437^*^	−0.481^**^	−0.435^*^
*Clostridiaceae 1*	0.427^*^	−0.003	−0.251	−0.076	0.017	−0.047
*Defluviitaleaceae*	−0.36	−0.407^*^	−0.362	−0.019	−0.049	0.011
*Enterococcaceae*	−0.452^*^	−0.551^**^	−0.406^*^	−0.246	−0.242	−0.199
*Peptococcaceae*	−0.404^*^	−0.383^*^	−0.124	0.128	−0.107	0.186
*Ruminococcaceae*	0.304	0.398^*^	0.307	0.177	0.258	0.174
*Staphylococcaceae*	0.382^*^	0.445^*^	0.527^**^	0.314	0.34	0.255
*Alcaligenaceae*	−0.377^*^	−0.546^**^	−0.295	−0.075	−0.208	−0.094
*Burkholderiaceae*	0.446^*^	0.297	0.285	0.259	0.426^*^	0.241
*Enterobacteriaceae*	−0.359	−0.523^**^	−0.336	−0.176	−0.14	−0.119
*Halomonadaceae*	0.659^**^	0.282	0.072	0.111	0.349	0.141
*Mollicutes RF9_norank*	0.237	0.445^*^	0.304	0.169	0.207	0.18

To the best of our knowledge, this is the first study to evaluate the therapeutic effects of ZT on RA by metabolomics and gut microbiota analysis. However, our study has several limitations: (1) We only detected small molecules with *m/z*-values under 500 using the GC-MS method, so it was difficult to comprehensively understand the overall metabolic process of RA. In future studies, we will apply the LC-MS technique to comprehensively monitor metabolic alterations in RA and evaluate the mechanism of ZT. (2) Our preliminary investigation found the correlation between gut microbiota and fecal metabolites, but additional experiments should be designed in future studies using special antibiotics administration to shift the composition of the gut microbiota and confirm the conclusion. (3) Our research was conducted based on rats; however, human samples should be collected from RA patients for metabolomics analysis to explore the biomarkers of the disease.

## Conclusion

In this study, serum and fecal metabolomics based on GC-MS, as well as 16S rRNA sequence analysis and pharmacodynamic evaluation (weight, foot swelling, arthritis score, inflammatory factor, and histopathology) were applied to determine the therapeutic effects of ZT on RA. The main objective of this study was to reveal the metabolic profiles of RA and the regulatory mechanisms of ZT. Several altered metabolites were identified, including l-asparagine, citric acid, l-isoleucine, and hypoxanthine. Furthermore, the pathway analysis of serum metabolomics demonstrated that the TCA cycle and fatty acid metabolism were disrupted in CIA rats. Disorders in purine metabolism were observed from the results of fecal metabolomics analysis. Sequence analysis results showed that the composition of gut microbiota was disturbed in CIA rats at the level of the family, and this could be rectified to some extent by ZT. In summary, this work indicated that metabolomics is a systematic approach for evaluating the therapeutic effects and mechanism of ZT.

## Ethics statement

This study was carried out in accordance with the recommendations of Animal Ethics Committee of Nanjing University of Chinese Medicine. The protocol was approved by the Animal Ethics Committee of Nanjing University of Chinese Medicine.

## Author contributions

LD and JS designed the experiments. LP, JS, WQ, and TX performed the experiments, analyzed the data and wrote the manuscript. AK and BG amended the paper.

### Conflict of interest statement

The authors declare that the research was conducted in the absence of any commercial or financial relationships that could be construed as a potential conflict of interest.
