# Supplementary material for: Integrated Serum and Fecal Metabolomics Study of Collagen-Induced Arthritis Rats and the Therapeutic Effects of the Zushima Tablet
Source: Front Pharmacol. 2018 Aug 14;9:891. doi: 10.3389/fphar.2018.00891 (PMC6102586; doi:10.3389/fphar.2018.00891)
Supplement: Supplementary file 1 [file Data_Sheet_1.PDF]

Supplementary Materials for
Integrated Serum and Fecal Metabolomics Study of Collagen-induced Arthritis rats and the Therapeutic Effects of Zushima Tablet

Jinjun Shan[1,2#*], Linxiu Peng[3#], Wenjuan Qian[3], Tong Xie[1,2], An Kang[3,4], Bei Gao[5], Liuqing Di[1,3,4*]

* **Correspondence:** diliuqing928@163.com (Liuqing Di); jshan@njucm.edu.cn (Jinjun Shan)

The PDF file includes:

Materials and Methods

Figure S1 EI-MS total ion chromatograph (TIC) of (A) serum and (B) fecal samples based on gas chromatography-mass spectrometry.

Figure S2 PCA score plots of metabolic profiling of QC and experimental samples in (A) serum and (B) fecal samples.

Table S1 The calibration curves, linear ranges, correlation coefficients (R2) of SCFAs

Table S2 The *P* value of correlation analysis for perturbed gut bacteria families and altered serum metabolites in day 36.

Table S3 The *P* value of correlation analysis for perturbed gut bacteria families and altered fecal metabolites in day 36.

Table S4. The *P* value of correlation analysis for perturbed gut bacteria families and short chain fatty acids in day 36.

Materials and Methods

Chemicals and materials

Daphnetin, daphnoretin, 7-hydroxycoumarin, luteoloside and kaempferol were all purchased from Sichuan Weikeqi Bio-tech Co., Ltd. (Chengdu, China). Apigenin was obtained from Chengdu Herbpurify Co.,LTD (Chengdu, China). All herb standard compounds were determined by HPLC and their purities were all higher than 98%.

Acetate was purchased from ROE Scientific (St. Louis, MO, USA). Propionate was purchased from Dr.Ehrenstorfer GmbH (Augsburg, Germany). Butyrate,

isobutyrate, valerate and isovalerate were all obtained from Tokyo Chemical Industry Co.,LTD (Tokyo, Japan). Pyridine and hexanoic acid-6,6,6-d3 were obtained from Sigma-Aldrich (St. Louis, MO, USA). Propyl chloroformate and 1-propanol were purchased from J&K® Scientific Co.,LTD (Beijing, China).

Quality evalution of ZT assessed by UHPLC-Q Exactive-Orbitrap-MS

ZT was removed with film coating and grinded. About 0.3 g ZT powder was weighed and placed into conical flask and 25 mL 85% methanol water solution was added in conical flask. The mixture was ultrasounded for 30min (the frequency was 50Hz, and the power was 500W). Eventually, the supernatant was obtained and injected into the UHPLC-Q Exactive-Orbitrap-MS.

The chromatographic separation was achieved based on Syncronis C18 column (100×2.1mm×1.7μm, Thermo). The injection volume used was 5μL. The column oven temperature was 40℃ and the mobile phase consisted of 0.1% formic acid in water (Solvent A) and acetonitrile (Solvent B) with a linear gradient elution: 0–2 min, 10% B; 2–20 min, 10–60% B; 20–21 min, 60– 90%B; 21–22 min, 90% B; 22–23 min, 90–10% B, and held for 5 min. The flow rate was 0.2 mL/min. The system was equipped with an ESI source, operated in negative ionization mode using the following parameters: capillary temperature was 300℃, sheath gas flow was 45 arbitrary units, auxiliary gas was 10 arbitrary units, source voltage was 3.5 kV. Data were collected from 100 m/z to 1000 m/z.

Quantification of SCFAs in fecal samples

100 mg fecal samples were spiked with 1 mL 0.005M NaOH solution and homogenized for 5 min, and the mixtures were centrifuged at 13,000 rpm for 10 min to obtain 0.6 mL supernatant. About 10 μL hexanoic acid-6,6,6-d_3 (internal standard, IS, 527μg/mL) were added in supernatant and the mixtures were vortexed for 5 min. Aliquots of 500 μL fecal samples with IS mixture were transferred into 10 mL glass centrifuge tubes, in addition, 300 μL water were added followed by 500 μL isopropanol/pyridine (3/2, v/v) and 100 μL propyl chloroformate for derivatization. The mixtures were vortexed and untrasounded for 1 min, and then 300 μL n-hexane were added for extracting SCFAs. After vortexing for 1 min, the mixtures were centrifuged at 2,000 rpm for 10 min and 200 μL supernatant was transferred into a 1.5 mL eppendorf tube. In addition, another 200 μL n-hexane were added for the second

step extraction. 400 μL supernatant in total were vortexed for injecting into GC-MS system for analysis.

The SCFAs were separated on TG-5MS capillary column (0.25 mm×30 m×0.25 μm, Thermo Fisher, San Jose, CA, USA) with a split ratio of 20:1. The injection volume was 1 μL. The gradient heating program was conducted as follows: 0–2 min, 50 ℃; 2–4 min, 50–70 ℃; 4–9 min, 70-85 ℃; 9-14 min, 85-110 ℃; 14-20 min, 110-290 ℃; 20-28 min, 290 ℃. Helium (99.999%) was used as the carrier gas, with a flow rate of 1.2 mL/min. TSQ 8000 was equipped with an Electron Ionization (EI) source. The ionization energy was 70 eV, the source temperature was 300 ℃, and the transfer line was held at 300 ℃. The GC-MS data were acquired after a solvent delay of 2.00 min, and the MS scan range was 30–600 m/z. The calibration curves were shown in **Table S1**.

Table S1 The calibration curves, linear ranges, correlation coefficients (R^2) of SCFAs

SCFAs	Calibration curves	R^2	Liner range (μg/mL)
Acetate	y = 0.0282x + 0.4725	0.9992	19.92-637.43
Propionate	y = 0.0144x + 0.5465	0.9994	25.07-401.14
Isobutyrate	y = 0.1963x + 0.1500	0.9995	1.60-51.25
Butyrate	y = 0.1482x + 0.3926	0.9991	3.13-200.43
Isovalerate	y = 0.1520x + 0.0962	0.9987	1.49-47.75
Valerate	y = 0.2437x + 0.0491	0.9996	1.57-100.50

Figure S1

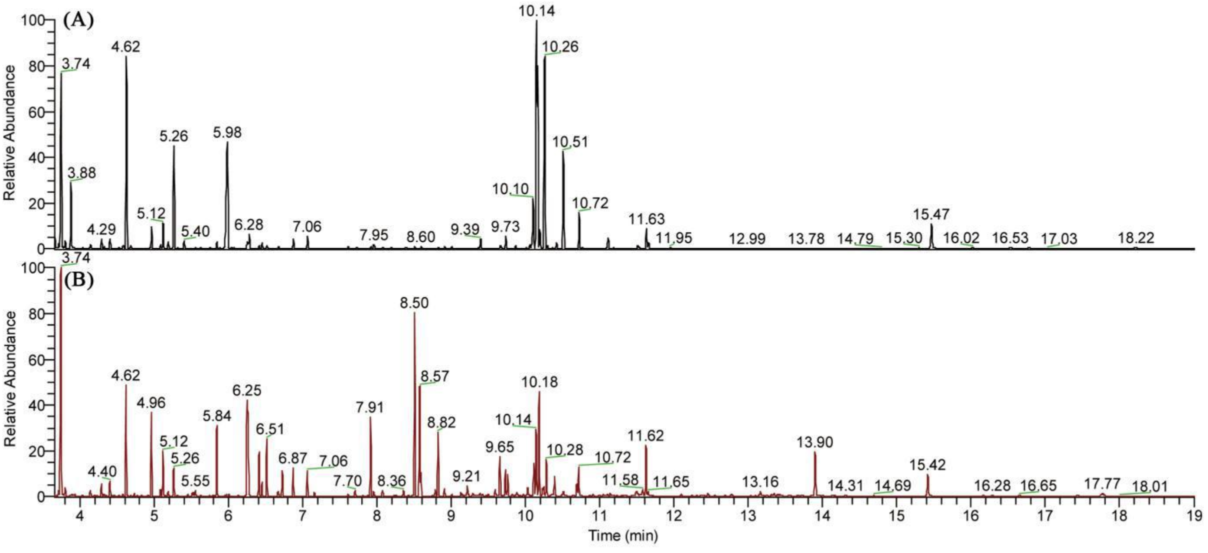

Figure S1 EI-MS total ion chromatograph (TIC) of (A) serum and (B) fecal samples based on gas chromatography-mass spectrometry.

Figure S2

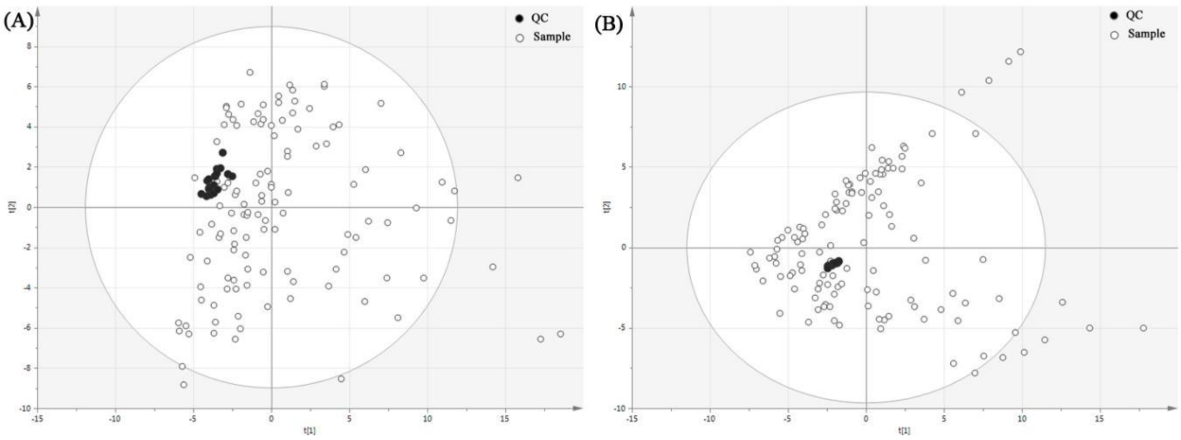

Figure S2 PCA score plots of metabolic profiling of QC and experimental samples in (A) serum and (B) fecal samples.

Table S2 The *P* value of correlation analysis for perturbed gut bacteria families and altered serum metabolites in day 36.

	Cholic acid	D-mannose	Palmitoleic acid	Spermidine	Threonic acid
Bifidobacteriaceae	0.276	0.01	0.271	0.063	0.006
Coriobacteriaceae	0.024	0.013	0.416	0.002	0.414
Corynebacteriaceae	0.004	0.125	0.126	0.009	0.678
Bacteroidaceae	0.001	0	0.088	0.032	0.663
Bacteroidales S24-7 group	0.024	0.024	0.047	0.17	0.623
Porphyromonadaceae	0.013	0.001	0.038	0.026	0.7
Gastranaerophilales_norank	0.001	0.026	0.045	0.02	0.252
Elusimicrobiaceae	0.417	0.482	0.412	0.021	0.841
Clostridiaceae 1	0.949	0.166	0.789	0.196	0.025
Defluviitaleaceae	0.01	0.891	0.003	0.003	0.143
Enterococcaceae	0.025	0.05	0.009	0.006	0.91
Peptococcaceae	0.1	0.387	0.083	0.399	0.199
Ruminococcaceae	0.007	0.035	0.404	0	0.084
Staphylococcaceae	0.003	0.073	0.011	0	0.195
Alcaligenaceae	0.031	0.022	0.053	0.022	0.256
Burkholderiaceae	0.982	0.44	0.757	0.021	0.156
Enterobacteriaceae	0.004	0.163	0.009	0.001	0.685
Halomonadaceae	0.644	0.007	0.061	0.054	0.036
Mollicutes RF9_norank	0.003	0.103	0.346	0.005	0.316

Table S3. The *P* value of correlation analysis for perturbed gut bacteria families and altered fecal metabolites in day 36.

	Adenine	Ethanolamine	Ferulic acid	L-Glutamine	L-Isoleucine	L-Leucine	L-Phenylalanine	L-Serine	Thymine	L-Tyrosine
Bifidobacteriaceae	0.035	0.106	0.047	0.101	0.143	0.389	0.153	0.041	0.929	0.041
Coriobacteriaceae	0.507	0.087	0.024	0.434	0.323	0.329	0.445	0.664	0.161	0.329
Corynebacteriaceae	0.201	0.087	0.785	0.654	0.643	0.992	0.633	0.43	0.047	0.814
Bacteroidaceae	0.458	0.072	0.224	0.992	0.302	0.392	0.773	0.798	0.154	0.55
Bacteroidales S24-7 group	0.276	0.086	0.412	0.855	0.278	0.352	0.635	0.943	0.33	0.528
Porphyromonadaceae	0.513	0.04	0.094	0.809	0.132	0.201	0.467	0.693	0.222	0.233
Gastranaerophilales_norank	0.165	0.009	0.107	0.128	0.032	0.144	0.568	0.728	0.012	0.536
Elusimicrobiaceae	0.642	0.014	0.018	0.71	0.912	0.582	0.93	0.588	0.814	0.542
Clostridiaceae 1	0.607	0.649	0.016	0.261	0.728	0.938	0.791	0.233	0.896	0.9
Defluviitaleaceae	0.072	0.08	0.526	0.138	0.007	0.02	0.044	0.344	0.057	0.099
Enterococcaceae	0.44	0.029	0.15	0.609	0.179	0.109	0.169	0.425	0.536	0.133
Peptococcaceae	0.005	0.365	0.929	0.274	0.175	0.488	0.773	0.858	0.062	0.687
Ruminococcaceae	0.083	0.143	0.003	0.092	0.121	0.164	0.423	0.211	0.592	0.178
Staphylococcaceae	0.022	0.004	0.341	0.988	0.405	0.777	0.948	0.993	0.046	0.674
Alcaligenaceae	0.254	0.001	0.425	0.73	0.261	0.388	0.266	0.71	0.567	0.136
Burkholderiaceae	0.121	0.373	0.035	0.26	0.414	0.711	0.8	0.494	0.496	0.555
Enterobacteriaceae	0.218	0.026	0.244	0.712	0.121	0.219	0.566	0.704	0.207	0.413
Halomonadaceae	0.182	0.158	0.003	0.03	0.031	0.124	0.232	0.055	0.167	0.15
Mollicutes RF9_norank	0.262	0.094	0.074	0.58	0.295	0.394	0.826	0.625	0.565	0.53

Table S4. The *P* value of correlation analysis for perturbed gut bacteria families and short chain fatty acids in day 36.

	Acetate	Propionate	Butyrate	Isobutyrate	Valerate	Isovalerate
Bifidobacteriaceae	0	0.061	0.457	0.604	0.077	0.404
Coriobacteriaceae	0.03	0.002	0.026	0.067	0.027	0.058
Corynebacteriaceae	0.232	0.166	0.015	0.119	0.256	0.244
Bacteroidaceae	0.078	0.016	0.069	0.228	0.08	0.192
Bacteroidales S24-7 group	0.013	0.012	0.026	0.064	0.009	0.108
Porphyromonadaceae	0.046	0.03	0.064	0.096	0.026	0.075
Gastranaerophilales_norank	0.014	0.046	0.282	0.941	0.47	0.707
Elusimicrobiaceae	0.7	0.066	0.036	0.018	0.008	0.018
Clostridiaceae 1	0.021	0.99	0.19	0.695	0.931	0.807
Defluviitaleaceae	0.055	0.028	0.053	0.921	0.802	0.953
Enterococcaceae	0.014	0.002	0.029	0.199	0.206	0.3
Peptococcaceae	0.03	0.041	0.52	0.51	0.582	0.335
Ruminococcaceae	0.109	0.032	0.105	0.36	0.176	0.368
Staphylococcaceae	0.041	0.016	0.003	0.097	0.071	0.181
Alcaligenaceae	0.044	0.002	0.12	0.697	0.279	0.629
Burkholderiaceae	0.015	0.118	0.134	0.174	0.021	0.208
Enterobacteriaceae	0.056	0.004	0.075	0.362	0.468	0.539
Halomonadaceae	0	0.138	0.71	0.566	0.064	0.466
Mollicutes RF9_norank	0.217	0.016	0.109	0.382	0.28	0.35
